# Behavioral Patterns, Rather Than Environmental Factors, Shape the Energy Balance of Wintering Chinese Mergansers (*Mergus squamatus*) in Huangshan

**DOI:** 10.1002/ece3.71225

**Published:** 2025-04-12

**Authors:** Chao Yu, Hao Zheng, Yu Luo, Mengxue Guo, Shiqi Wang, Xuanshuo Qi, Qun Li, Zhonghai Lv

**Affiliations:** ^1^ College of Life and Environmental Sciences Huangshan University Huangshan China; ^2^ Environment Conservation Research Centre of Xin'an River Basin Huangshan University Huangshan China; ^3^ Department of Resources Conservation Southern Anhui National Wildlife Rescue Center Xiuning City China; ^4^ Academy of Environmental Planning & Design, co., ltd. Nanjing University Nanjing City China

**Keywords:** Chinese merganser, energy strategy, environmental factor, Huangshan, wintering behavior

## Abstract

Animal energy intake and expenditure strategies in response to environmental fluctuations have been widely studied. Optimal foraging theory (OFT) is the dominant framework in this field; however, studies addressing the OFT in endangered waterbird species are lacking. To address this gap in our knowledge, we collected behavioral data and investigated habitat variables that influence the energy intake and expenditure of the endangered Chinese merganser ( *Mergus squamatus* ) in the Changjiang, Shuaishui, and Jianjiang Rivers in Huangshan, Anhui Province, China, from January to March 2023. The results revealed a correlation between net energy, energy intake, and energy expenditure rates. Successful foraging frequencies increased net energy intake and energy intake rates and reduced the feeding time. Furthermore, running on water, flying, diving, and average fish weight increased energy expenditure rates, whereas successful foraging frequencies, vigilance, resting, fish biomass, river width, and eye‐submerging decreased energy expenditure rates. Chinese mergansers adjusted behavioral time allocations to regulate energy intake, energy expenditure, and net energy intake rates. The net energy and energy intake rates were independent of environmental factors, excluding energy expenditure rates and average fish weight, fish biomass, and river width. The behaviors influencing the energy balance were modified in response to environmental factors. However, environmental factors did not affect the energy levels through behaviors. These results elucidate the energy intake, expenditure, and balance strategies used by Chinese mergansers in response to variations in their wintering habitats. They provide valuable insights for conserving and managing habitats critical to the survival of Chinese mergansers and other waterbird species.

## Graphical abstract

Our findings have elucidated the energy intake, expenditure, and balance strategies used by Chinese mergansers in response to variations in their wintering habitats, providing valuable insights for conserving and managing habitats critical to the survival of Chinese mergansers and other waterbird species.

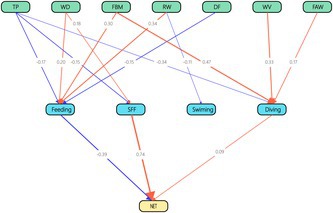

## 1 Introduction

Energy balance strategies are crucial to the life history of animals, such as survival and migration (Londoño et al. 2014; Mcnab 2003; Schmaljohann and Eikenaar 2017; Weimerskirch et al. 2001). Researchers have conducted in‐depth investigations in this field (Louzao et al. 2014), and in particular, the optimal foraging theory (OFT) has been widely evaluated (Pyke et al. 1977; Pyke 2019; Werner and Hall 1974). The OFT is a behavioral ecology model that predicts animal behavior during foraging. Animals maximize fitness by adopting foraging strategies that maximize net energy gain—obtaining the greatest energy benefit while minimizing cost (Kay 2002; Pyke 1984). The model‐building process involves identifying foragers' currency, constraints, and appropriate decision rules (Stephens and Krebs 1986). In this context, animals obtain energy from food, and the currency is the net energy gain per unit of time (Sinervo 1997). This currency represents the difference between the energy gained from food and expenditure during the search and capture process (constraints). Constraints are factors that limit the ability of a forager to maximize currency (Sinervo 1997).

Currency, energy gain, and expenditure are primarily limited by environmental factors (Yu et al. 2020; Zhang et al. 2019; Liu et al. 2023b). Animals typically optimize their energy gain and currency through energy balance strategies, which reflect the broader principles observed across all organisms, including waterbirds (Urban 2007). Energy gain by wintering swan geese and swans is influenced by food quality, quantity, and foraging investment (Chen et al. 2019; Yu et al. 2020), whereas currency is increased by food size, biomass, foraging time, and success (Clausen et al. 2018; Yu et al. 2019a, 2019b). Constraints affecting foraging include lethal and threatening disturbances (Nolet et al. 2016; Yu et al. 2020), high water depth and velocity, changes in habitat safety, food resource availability, and temperature. This can directly or indirectly affect the gain, currency, and expenditure due to waterbirds (such as Spot‐billed ducks, swans, and others) adjusted behavioral patterns including foraging, vigilance, resting, and so on (Yu et al. 2024; Zeng et al. 2013; Nolet et al. 2006; Zanghi et al. 2024). For endangered waterbirds vulnerable to disturbance, their behaviors, energy intake, expenditure, and balance are constrained by environmental factors, which remain unclear, and research on their response patterns is limited (Liu et al. 2023b; Lu et al. 2021; Shao and Chen 2017).

**FIGURE 1:**
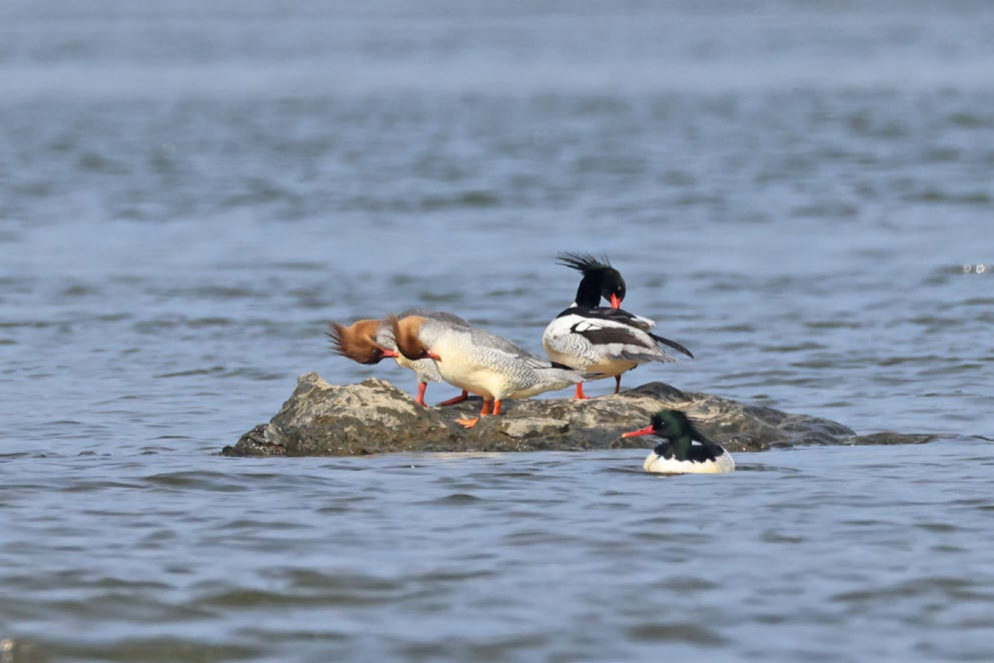
The Chinese mergansers in the rivers of Huangshan. The left two are females and the right two are males, respectively. Credit: Chao Yu.

Chinese mergansers ( *Mergus squamatus* ) (Figure 1) are a member of the Anatidae, Anseriformes. They are a first‐class key protected waterbird in China and are listed as “Endangered” (BirdLife International 2017), because the population comprises approximately 2400‐4500 individuals worldwide. The rivers in Huangshan are important wintering waterbird habitats on the East Asian–Australasian Flyway, particularly for Chinese mergansers. The water in these rivers is relatively clear, and the riverbed is flat, with dense plants beside the shore that attract Chinese mergansers and residents. The wintering period is an important part of the annual life history of waterbirds (Wang et al. 2016) and is a critical life stage for migration. During winter, the scarcity of food resources and changes in environmental conditions can directly affect the energy balance of waterbirds and are directly related to their survival. The potential conflict between Chinese mergansers and humans affects the behavior, energy intake, expenditure, and surplus of Chinese mergansers. However, studies on the energy balance of Chinese mergansers during the wintering period, specifically the relationship between energy balance and habitat factors, are limited (Liu et al. 2023b; Shao and Chen 2017).

To address the gaps in our knowledge, we observed and documented the time allocation of various behaviors exhibited by Chinese mergansers in a dynamic environment. This study focused on energy intake, expenditure, and net intake rates under different environmental conditions during the winter season. Concurrently, we analyzed the characteristics associated with wintering Chinese mergansers. We hypothesized that: (1) Chinese mergansers adjusted their energy intake as energy expenditure changed to maximize net energy intake; (2) Chinese mergansers regulated behavioral patterns, increased energy intake, and reduced expenditures in response to different human disturbances, water depth, average fish weight, and fish biomass; (3) environmental factors influenced energy intake, expenditure, and net energy intake rate via behavioral changes.

## 2 Methods

### 2.1 Study Sites

**FIGURE 2:**
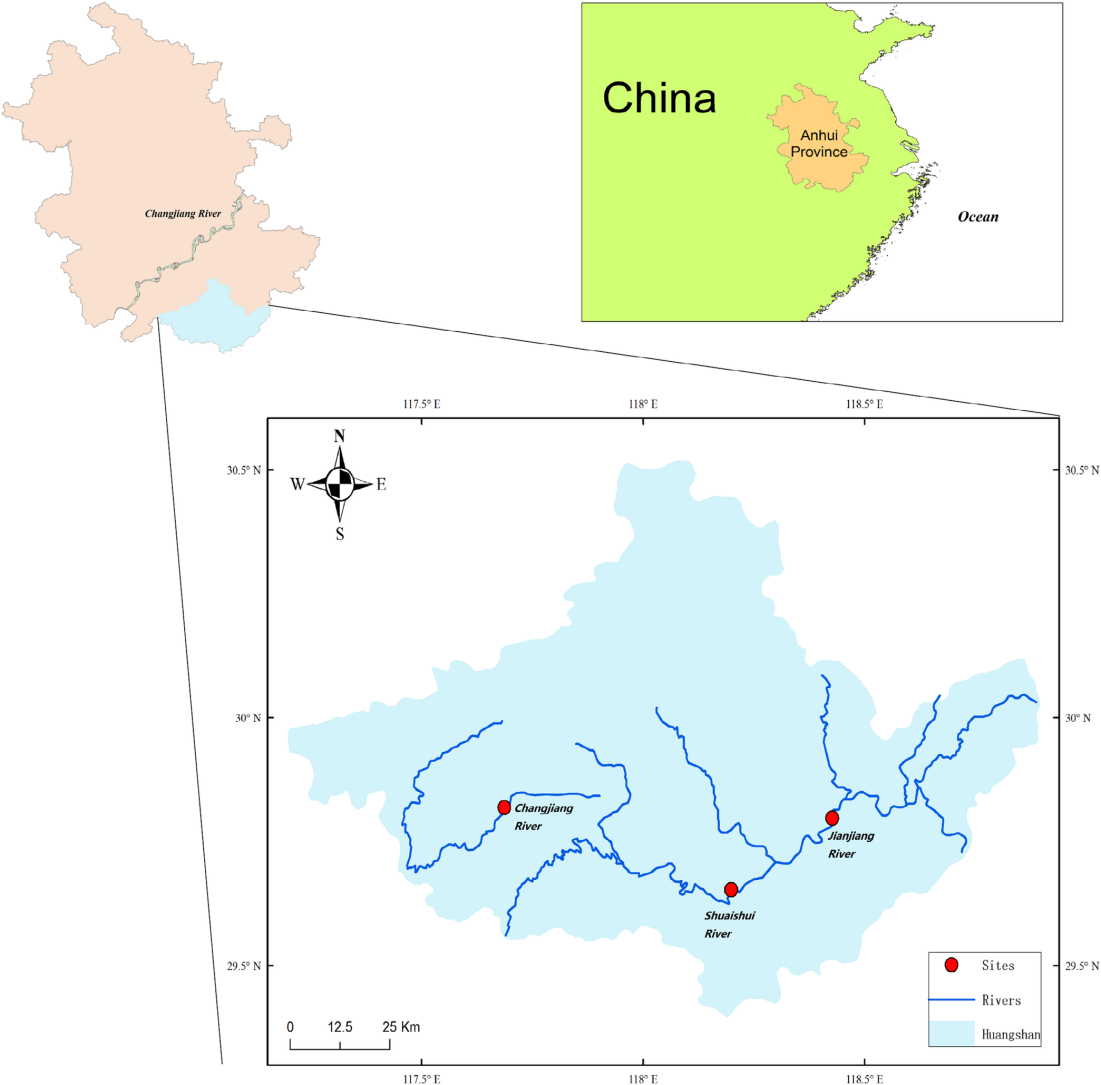
Habitats of wintering Chinese mergansers in Huangshan, Anhui Province, China. Blue lines and red dots indicate the rivers and study sites.

We collected behavioral and environmental data for the endangered Chinese merganser in the Changjiang, Jianjiang, and Shuaishui Rivers in Huangshan, Anhui Province, China (Figure 2). Chinese mergansers inhabit a series of sites along the three rivers. The Changjiang River is relatively narrow at approximately 50 m, with turbulent water flow and dense bamboo forests on both sides. The Jianjiang River is wide ( > 150 m), with gentle water flow and grassland landscapes with considerable human activity on both sides. The Shuaishui River is wider than the Changjiang River but narrower than the Jianjiang River, with bush and tea on both sides. Residents live near these rivers and engage in agriculture, indicating that human activities substantially impact Chinese mergansers' habitats. During the observation period, more than 100 individuals of Chinese mergansers wintered at the study rivers. The fish species that we caught in the studied rivers included *Acrossocheilus fasciatus, Misgurnus anguillicaudatus, Carassius auratus*, and *Gobius* spp.

### 2.2 Behavior and Environmental Factors

The Chinese merganser breeds in the Changbai Mountains in northeast China and Russia and migrates from its breeding grounds to wintering sites in Huangshan during autumn (BirdLife International 2017). This study was conducted during the Chinese mergansers wintering period. We videoed the behaviors of Chinese mergansers using a PhotoScope (Victory PhotoScope 85 T*FL, 15–45×, Jena, Germany) in a camouflage tent. A random foraging individual was recorded for approximately 5 min using a focal animal method at intervals of 30 min (Altmann 1974; Yu et al. 2024). The focal animal method is a sampling technique in which the behavior of one individual is observed and recorded for a specified amount of time (Paul and Patrick 2007). They were observed from 7:30 to 17:30 or the time of their departure. In total, 87 focal animal samples were collected from January to March 2023.

A disturbance source appeared at a fixed point and on the road. The observer followed the disturbance source while it was within the field of vision of the videoed mergansers, the observer, the disturbance source, and the videoed Chinese mergansers in a straight line. A laser rangefinder telescope (Zeiss Victory Scope, 10 × 42, Jena, Germany) was used to determine the shortest distance between the videoed merganser and the disturbance sources (electronic bikers, washing, recreational walking, and pole or net fishing appearing at the waterbird eye horizon). The magnitudes of the various disturbances were comparable, with the primary distinction being their duration within the merganser's visual field, attributable to their differing speeds. During the focal sampling, we documented and analyzed solely the duration of the disturbances, rather than their specific types (Yu et al. 2019a ). The disturbance number was recorded: the quantity of disturbance source at the Chinese merganser's eye horizon in a focal animal sample.

We recorded air temperature (from http://www.weather.com.cn/) while observing Chinese merganser behavior. The active range of Chinese mergansers is too large because it can reach hundreds of square meters in the videoed time. The actual measured water conditions will scare them away, and this is against animal welfare. Meanwhile, Chinese mergansers have been reported to inhabit a slow water environment (Yi et al. 2019). At high velocities, the shoreside is an important area for their activities, whereas, at low velocities, mergansers occur at the shoreside and other areas of the river when the differences in water velocities are small. Therefore, we used a flowmeter (Xiangruide, LS1206b) and ruler, which were placed 5 m from a fixed shore point to measure relative water velocity and water depth, thus enabling relatively accurate calculations of water velocity and level fluctuations at the merganser's foraging areas. It was difficult to confirm the specific species of fish consumed by the mergansers due to the far distance from the videoed individual, the random sample of fish by creels with holes that allow fish < 16 cm access. Fish biomass was collected from 12 creels in the study area. The body length and weight of the fish were determined, and the fish biomass was 0.9–15.3 g/m^2^.

### 2.3 Statistical Analysis

We classified behaviors included foraging (diving, head‐dipping, eye‐submerging), feeding, resting, comfort (splashing and preening), vigilance, swimming, flying (Owen and Black 1990), and running on water (wing beating and swimming while chasing fish on water) (Liu et al. 2023a). The allocation time, percentage, and frequency of behaviors for each focal animal sample were obtained using a PIPI Player (version 3.4.0, Ku6 Media Co. Ltd., Beijing, China) to display and analyze the videos with frame‐by‐frame viewing on the computer.

Chinese mergansers eat common fish of Cypriniformes and Perciformes in their wintering grounds (Liu et al. 2023b), and the fat, protein, water content, and density of energy of the fish are similar (Liang et al. 2015). Meanwhile, the difficulty in determining the specific fish species eaten by videoed mergansers leads researchers to use an average fish energy content of 4000 J/g (Gremillet et al. 1995). With an assimilation efficiency of 80% (Feltham 1995), the energy intake in each focal animal sample can be calculated as follows: energy intake (J) = average fish weight (g) × 4000 (J/g) × 80% × successful foraging frequency.

We were unable to determine the weight of individual Chinese mergansers because they are under strict protection in China and are difficult to capture. Based on the body mass (male 1125–1400 g, female 870–1100 g) of *M. squamatus* (Kear and Hulme 2005), the basal metabolic rate (BMR) was determined using the Aschoff‐Pohl equation (Aschoff and Pohl 1970) as follows: BMR = 307.5 body mass^0.734^, male 365.00 kJ/day, and female 304.12 kJ/day (Liu et al. 2023b). Because subadults and females were difficult to identify, we averaged the BMR of females and males to 3.872 J/s. The total energy expenditure was calculated by multiplying the time allocation, BMR, and multiples of each behavior in a focal animal sample as follows:

(1) $$\text{EE}=\sum_{x=1}^{m}x_{\text{multiple}}\times \mathrm{BMR}\times x_{\text{time allocation}}$$

where EE is the sum of activity energy expenditure of behaviors in a focal animal sample, *x*_multiple_ is the multiple of BMR for behavior *x*, and *x*_time allocation_ is the time allocation of behavior *x* in a focal animal sample; *m* is the number of behavior types of the focal animal sample.

Foraging included three types, namely, diving, head‐dipping, and eye‐submerging, and their BMR multiples were 3.2 (Newson and Hughes 1998), 2.5 (Jónsson and Afton 2006), and 2.0 (Jónsson and Afton 2006), respectively, while the BMR multiple for vigilance was 2.0 (Newson and Hughes 1998) and that for running on water (its parameter was the average of flight and swimming) was 7.4. The BMR multiples for resting, swimming, comfort, flying, and feeding were 1.2, 2.2, 2.1, 12.5, and 1.7, respectively (Jones et al. 2014; Wooley and Owen 1977). .

The net energy intake differs between each focal animal sample's energy intake and total energy expenditure. We calculated the rates of energy intake, energy expenditure, and net energy intake by dividing the focal animal sample's time by energy intake, energy expenditure, and net energy intake.

We developed a comparative analysis of energy intake, energy expenditure, net energy intake rates, the percentage of time allocated to various behaviors, and environmental variables (average fish weight, fish biomass, river width, water velocity, temperature, water depth, disturbance number, disturbance duration, and disturbance distance) using Student's *t*‐test, given that the data exhibited normal distribution and homoscedasticity. In cases where these assumptions were not met, nonparametric tests were used. In addition, we assessed the relative correlations among energy intake, energy expenditure, net energy intake rates, and the percentage of time allocated to behaviors in relation to environmental conditions using Pearson correlation tests to obtain the predictor variables that can be entered in the Generalized linear models (GLMs) in the next step.

Energy intake, energy expenditure, and net energy intake rates were analyzed against environmental and behavior predictors with which they had relationships. Most of the data had a right‐skewed distribution; therefore, we log (x + 1) transformed the primary behaviors with energy balance (diving, eye‐submerging, vigilance, SFF, feeding, and swimming), energy intake, and cube‐root transformed the net energy intake rates when they were used as response variables. Since the combined effects of behavioral coefficients of BMR and allocation times on energy content together. Energy intake and net energy intake rates fitted the predictors of successful foraging frequency, feeding, swimming, and diving time percentage. Energy expenditure fitted the predictors of running on water, flying, diving, average fish weight, successful foraging frequency, vigilance, resting, fish biomass, river width, and eye‐submerging. Primary behaviors with energy balance (diving, eye‐submerging, vigilance, SFF, feeding, and swimming) relatively fitted the predictors that had a relationship with them. The Generalized linear model was used to analyze the relationship between the response variable and the predictors. The Gaussian family and “identity” link function were used in the model. We used pseudo R^2^ to evaluate model goodness of fit: 0.2–0.4 indicates good model fit, 0.1–0.2 general model fit, and < 0.1 poor model fit (McKelvey and Zavoina 1975). The likelihood ratio test was used to compare the fitted model with the null model. Before this, we ensured no collinearity among the predicted variables, as indicated by correlation coefficients < 0.8 (Kim 2019).

An assessment was conducted of the direct and indirect influences of environmental factors and behavior changes on energy intake, energy expenditure, and net energy intake rates. Structural equation modeling (SEM) was conducted using the maximum likelihood estimation method within the “lavaan” package of R. Environmental factors and behavioral time percentages were considered observed variables and mediators, whereas energy intake, energy expenditure, and net energy intake rates were considered endogenous variables. Optimal model fitting involved the *χ*2 test with *p* > 0.05, root mean square error of approximation (RMSEA) < 0.06, standardized root mean square residual (SRMR) < 0.09, and the comparative fit index (CFI) should be ≥ 0.95 (Hu and Bentler 1999).

We used R (version 4.1.2; R Core Team, 2023), and IBM SPSS Statistics 26 for data analysis and graphing. The data are presented as mean ± standard deviation. A significance threshold of *p* < 0.05 was established for all statistical analyses.

## 3 Results

This study included 87 focal Chinese mergansers from three rivers (Additional file 1). The net energy intake rates were positively correlated with the energy intake rates (*r* = 1.000, *p* = 0.000) and energy expenditure rates (*r* = 0.270, *p* = 0.011); meanwhile, the energy intake and energy expenditure rates had a positive relationship (*r* = 0.283, *p* = 0.008). This indicates that mergansers can achieve a sufficient energy surplus by increasing their energy intake and expenditure. The frequency of vigilance behavior (23.701 ± 18.437) was the highest; however, diving time (104.976 ± 79.050 s) and percent (34.729% ± 26.073%) were higher than other behaviors in samples (Tables 1, 2, 3).

**TABLE 1:** Behavior frequency of Chinese mergansers in different wintering sites.

Wintering sites	Resting	Running on water	Head‐dipping	Diving	Flying	Eye‐submerging	Swimming	Vigilance	Comfort	Feeding	Success	Failure
Changjiang River (*n* = 12)	3.250 ± 2.491	1.167 ± 2.517	0.000 ± 0.000	0.000 ± 0.000	0.083 ± 0.289	15.583 ± 13.007	7.333 ± 7.266	22.083 ± 18.788	2.750 ± 3.911	0.250 ± 0.622	0.167 ± 0.577	15.417 ± 12.845
Shuaishui River (*n* = 34)	0.029 ± 0.172	0.471 ± 0.992	0.206 ± 0.538	7.265 ± 3.808	0.059 ± 0.343	11.294 ± 18.724	6.941 ± 6.980	29.324 ± 23.189	1.559 ± 2.631	4.588 ± 4.573	1.118 ± 1.122	17.588 ± 17.024
Jianjiang River (*n* = 41)	0.000 ± 0.000	0.683 ± 2.161	0.024 ± 0.156	6.146 ± 4.108	0.073 ± 0.264	6.634 ± 7.245	6.951 ± 3.146	19.512 ± 12.021	2.293 ± 2.704	2.098 ± 2.107	0.902 ± 1.338	11.878 ± 7.567
Total (*n* = 87)	0.460 ± 1.437	0.667 ± 1.847	0.092 ± 0.362	5.736 ± 4.360	0.069 ± 0.297	9.690 ± 13.816	7.000 ± 5.484	23.701 ± 18.437	2.069 ± 2.864	2.816 ± 3.542	0.885 ± 1.205	14.598 ± 12.888

**TABLE 2:** Behavior time and percent of Chinese mergansers in different wintering sites.

Wintering sites	Item	Resting time (s)	Running on water time (s)	Head‐dipping time (s)	Diving time (s)	Flying time (s)	Eye‐submerging time (s)	Swimming time (s)	Vigilance time (s)	Comfort time (s)	Feeding time (s)	Total time (s)
Changjiang River (*n* = 12)	Time (s)	41.787 ± 32.553	6.403 ± 14.582	0.000 ± 0.000	0.000 ± 0.000	1.879 ± 6.508	47.266 ± 48.159	91.216 ± 90.792	79.291 ± 53.439	11.489 ± 18.522	0.798 ± 1.887	280.128 ± 34.356
	Percent (%)	15.043 ± 10.907	2.576 ± 5.970	0.000 ± 0.000	0.000 ± 0.000	0.631 ± 2.185	16.452 ± 16.174	31.211 ± 31.290	29.772 ± 22.443	4.037 ± 6.266	0.278 ± 0.663	
Shuaishui River (*n* = 34)	Time (s)	0.186 ± 1.081	1.793 ± 4.572	1.097 ± 3.316	127.601 ± 81.885	1.823 ± 10.629	37.872 ± 63.158	50.647 ± 43.593	57.039 ± 32.114	12.115 ± 33.063	11.913 ± 11.686	302.085 ± 8.207
	Percent (%)	0.063 ± 0.368	0.587 ± 1.487	0.371 ± 1.134	42.207 ± 27.067	0.622 ± 3.627	12.563 ± 20.918	16.764 ± 14.317	18.883 ± 10.621	4.001 ± 10.900	3.939 ± 3.855	
Jianjiang River (*n* = 41)	Time (s)	0.000 ± 0.000	3.016 ± 10.402	0.211 ± 1.352	116.939 ± 63.370	1.527 ± 7.887	33.528 ± 47.508	78.470 ± 50.767	45.576 ± 21.840	15.128 ± 22.237	6.747 ± 9.202	301.141 ± 10.730
	Percent (%)	0.000 ± 0.000	0.992 ± 3.404	0.077 ± 0.495	38.693 ± 20.779	0.505 ± 2.603	11.123 ± 15.779	26.127 ± 16.832	15.190 ± 7.390	5.061 ± 7.424	2.232 ± 2.994	
Total (*n* = 87)	Time (s)	5.836 ± 18.580	3.005 ± 9.367	0.528 ± 2.299	104.976 ± 79.050	1.691 ± 8.816	37.120 ± 53.831	69.355 ± 56.806	54.706 ± 33.303	13.448 ± 26.380	7.945 ± 10.309	298.611 ± 16.907
	Percent (%)	2.100 ± 6.510	1.052 ± 3.348	0.181 ± 0.795	34.729 ± 26.073	0.568 ± 2.969	12.421 ± 17.896	23.169 ± 19.107	18.645 ± 12.494	4.505 ± 8.748	2.630 ± 3.384	

**TABLE 3:** Behavior energy expenditure rates of Chinese mergansers in different wintering sites.

Wintering sites	Resting energy expenditure (J/s)	Running on water energy expenditure (J/s)	Head‐dipping energy expenditure (J/s)	Diving energy expenditure (J/s)	Flying energy expenditure (J/s)	Eye‐submerging energy expenditure (J/s)	Swimming energy expenditure (J/s)	Vigilance energy expenditure (J/s)	Comfort energy expenditure (J/s)	Feeding energy expenditure (J/s)
Changjiang River (*n* = 12)	194.161 ± 151.253	183.455 ± 417.816	0.000 ± 0.000	0.000 ± 0.000	90.928 ± 314.982	366.025 ± 372.942	777.013 ± 773.405	614.032 ± 413.831	93.420 ± 150.607	5.249 ± 12.423
Shuaishui River (*n* = 34)	0.862 ± 5.025	51.359 ± 131.009	10.617 ± 32.096	1581.022 ± 1014.588	88.230 ± 514.467	293.282 ± 489.097	431.435 ± 371.346	441.711 ± 248.694	98.509 ± 268.843	78.415 ± 76.924
Jianjiang River (*n* = 41)	0.000 ± 0.000	86.417 ± 298.045	2.044 ± 13.086	1448.919 ± 785.180	73.925 ± 381.720	259.637 ± 367.904	668.438 ± 432.454	352.938 ± 169.128	123.007 ± 180.812	44.409 ± 60.572
Total (*n* = 87)	27.118 ± 86.328	86.101 ± 268.386	5.112 ± 22.250	1300.695 ± 979.466	81.861 ± 426.710	287.459 ± 416.870	590.792 ± 483.900	423.644 ± 257.899	109.352 ± 214.504	52.297 ± 67.860

### 3.1 Net Energy Intake, Energy Intake, Expenditure Rates and Environment Factors at Different Sites

Net energy intake, energy intake, and energy expenditure rates of the Chinese mergansers varied at the different sites. Net energy intake rates were highest in the Jianjiang River, with no significant difference compared with the Changjiang River (*Z* = −0.383, *p* = 0.702) and Shuaishui River (*Z* = −0.511, *p* = 0.609). Energy intake rates in the Jianjiang River were significantly higher than those in the Changjiang River (*Z* = −2.267, *p* = 0.023), whereas no significant difference was found between the Jianjiang and Shuaishui Rivers (*Z* = −0.418, *p* = 0.676).

The energy expenditure rate was the highest in the Changjiang River, with no difference between the Shuaishui and Jianjiang Rivers (*Z* = −0.032, *p* = 0.957) and significant differences among the remaining sites.

Average fish weight, fish biomass, river width, and water velocity were highest at Jianjiang River and showed variation among the three rivers. The only exceptions were for water width, which was similar between Jianjiang and Shuaishui Rivers, and temperature, which was similar between Changjiang and Shuaishui Rivers (Table 4).

**TABLE 4:** Net energy intake, energy intake, and energy expenditure rates of Chinese mergansers in different wintering sites.

Wintering sites	Net energy intake rates (J/s)	Energy intake rates (J/s)	Energy expenditure rates (J/s)	Average fish weight (g)	Fish biomass (g/m^2^)	Temperature (°C)	River width (m)	Water depth (m)	Water velocity (m/s)	disturbance number	Disturbance duration(s)	Disturbance distance(m)
Changjiang River (*n* = 12)	−6.146 ± 8.974	2.500 ± 8.659	8.617 ± 0.261	1.553 ± 0.592	5.200 ± 0.000	8.667 ± 1.497	40.000 ± 0.000	0.9000 ± 0.000	0.120 ± 0.000	0.000 ± 0.000	0.000 ± 0.000	−
Jianjiang River (*n* = 41)	74.382 ± 153.538	84.940 ± 153.900	8.377 ± 0.362	2.768 ± 1.167	26.712 ± 27.770	11.324 ± 5.503	159.735 ± 27.390	0.672 ± 0.197	0.227 ± 0.096	0.677 ± 1.093	95.177 ± 130.965	235.000 ± 83.643
Shuaishui River (*n* = 34)	27.864 ± 39.490	38.011 ± 39.688	8.417 ± 0.498	3.213 ± 0.240	20.983 ± 8.280	9.317 ± 6.479	127.144 ± 48.308	0.679 ± 0.307	0.139 ± 0.092	1.463 ± 2.346	90.220 ± 116.335	285.350 ± 108.416
Total (*n* = 87)	45.327 ± 111.613	55.240 ± 112.056	9.913 ± 1.600	2.810 ± 0.945	21.045 ± 19.379	10.012 ± 5.709	127.862 ± 53.407	0.706 ± 0.255	0.171 ± 0.098	0.954 ± 1.817	79.713 ± 117.941	264.620 ± 100.778
Changjiang and Shuaishui River	*Z* = −2.201 *p* = 0.028	*Z* = −1.576 *p* = 0.115	*Z* = −3.262 *p* = 0.001	*Z* = 2.824 *p* = 0.000	*Z* = 3.047 *p* = 0.000	*Z* = 1.040 *p* = 0.229	*Z* = 3.047 *p* = 0.000	*Z* = 2.155 *p* = 0.000	*Z* = 2.155 *p* = 0.000	*Z* = 1.486 *p* = 0.024	Z = 1.486 *p* = 0.024	−
Changjiang and Jianjiang River	*Z* = −0.383 *p* = 0.702	*Z* = −2.267 *p* = 0.023	*Z* = −2.508 *p* = 0.012	*Z* = 2.19 *p* = 0.000	Z = 1.664 *p* = 0.008	*Z* = 1.664 *p* = 0.008	*Z *= 2.978 *p* = 0.000	*Z* = 2.102 *p* = 0.000	*Z* = 2.978 *p* = 0.000	*Z* = 1.226 *p* = 0.99	*Z* = 1.226 *p* = 0.99	−
Shuaishui and Jianjiang River	*Z* = −0.511 *p* = 0.609	*Z *= −0.418 *p* = 0.676	*Z* = −0.032 *p* = 0.957	*Z* = 1.398 *p* = 0.040	*Z* = 2.409 *p* = 0.000	*Z* = 1.092 *p* = 0.184	*Z* = 3.155 *p* = 0.000	*Z* = 2.524 *p* = 0.000	*Z* = 2.409 *p* = 0.000	*Z *= 0.588 *p* = 0.880	*Z* = 0.594 *p* = 0.572	Z = 1.107 *p* = 0.172

*Note:* −, no data or results between two variables.

### 3.2 Relationship Between Energy Expenditure Rates and Environmental Factors

The energy expenditure rates of Chinese mergansers were 9.913 ± 1.600 J/s. Energy expenditure rates were negatively correlated with resting (*r* = −0.392, *p* = 0.000), eye‐submerging (*r* = −0.331, *p* = 0.002), and vigilance (*r* = −0.249, *p* = 0.002), and positively correlated with running on water time (*r* = 0.299, *p* = 0.005), flying (*r* = −0.612, *p* = 0.000), diving time (*r* = 0.508, *p* = 0.000), fish biomass (*r* = 0.244, *p* = 0.023), successful foraging frequency (*r* = 0.294, *p* = 0.006), average fish weight (*r* = 0.326, *p* = 0.002), and river width (*r* = 0.229, *p* = 0.033). Diving increased with average fish weight (*r* = 0.351, *p* = 0.001), successful foraging frequency (*r* = 0.362, *p* = 0.001), fish biomass (*r* = 0.554, *p* = 0.000), decreased temperature (*r* = −0.384, *p* = 0.000), and water velocity (*r* = −0.231, *p* = 0.032), which together significantly led to changes in diving time. The model was significantly better than the null model (*χ* ^2^ = 76.540, *p* = 0.000), and the Pseudo *R* ^2^ was 0.133 (Table 5). Eye‐submerging increased with increased water velocity (*r* = 0.299, *p* = 0.005) and temperature (*r* = 0.427, *p* = 0.000) and decreased fish biomass (*r* = 0.293, *p* = 0.006), but only water velocity significantly led to changes in eye‐submerging time. The model was similar to the null model (*χ* ^2^ = 0.601, *p* = 0.125), (Table 5). Vigilance had a negative relationship with average fish weight (*r* = −0.335, *p* = 0.002) and river width (*r* = −0.272, *p* = 0.011); however, only average fish weight led to changes in vigilance time. The model was significantly better than the null model (*χ* ^2^ = 3.265, *p* = 0.007) and Pseudo *R* ^2^ was 0.061 (Table 5). Resting increased with increased water depth (*r* = 0.243, *p* = 0.023) but decreased with disturbance duration (*r* = −0.220, *p* = 0.004), river width (*r* = −0.528, *p* = 0.000), average fish weight (*r* = −0.438, *p* = 0.000), and fish biomass (*r* = −0.256, *p* = 0.000). Successful foraging frequency had a positive relationship with water depth (*r* = 0.242, *p* = 0.024) and feeding (*r* = 0.431, *p* = 0.001) but decreased with increased temperature (*r* = −0.222, *p* = 0.039); these variables did not significantly lead to changes in successful foraging frequency time. The model was significantly better than the null model (*χ* ^2^ = 2.066, *p* = 0.026), and the Pseudo *R* ^2^ was 0.051 (Table 5).

**TABLE 5:** Summary of the final models for Chinese mergansers' behaviors and energetics.

Response variables	Predictor variables	Estimate	Std. Error	*t*‐value	*p* (t)	(*χ* ^2^)	*p* (*χ* ^2^)	*R* ^2^
Log (Diving+1)	Intercept	0.190	0.557	0.342	0.733	76.540	0.000	0.133
	FAW	0.595	0.157	3.791	0.000			
	FBM	0.029	0.010	3.056	0.003			
	TP	−0.080	0.038	−2.109	0.038			
	WV	7.377	2.156	3.422	0.001			
Log (Eye‐submerging+1)	Intercept	−0.016	0.124	−0.127	0.899	0.601	0.125	0.111
	FBM	0.000	0.002	0.033	0.974			
	TP	−0.012	0.010	−1.226	0.224			
	WV	1.274	0.571	2.230	0.028			
Log (Vigilance+1)	Intercept	2.979	0.263	11.346	0.000	3.265	0.007	0.061
	FAW	−0.168	0.066	−2.558	0.012			
	WD	0.430	0.244	1.764	0.081			
Log (SFF+1)	Intercept	0.418	0.248	1.687	0.095	2.066	0.026	0.051
	TP	−0.018	0.011	−1.654	0.102			
	WD	0.314	0.247	1.275	0.206			
Log (Feeding+1)	Intercept	0.596	0.244	2.445	0.017	23.614	0.000	0.228
	RW	0.009	0.002	4.353	0.000			
	FBM	0.002	0.006	0.421	0.675			
	WV	0.714	1.255	0.569	0.571			
	TP	−0.084	0.022	−3.795	0.000			
	DF	−0.085	0.039	−2.177	0.032			
Log (Swimming+1)	Intercept	2.998	0.180	16.667	0.000	4.381	0.065	0.052
	FBM	−0.012	0.006	−1.847	0.068			
EE	Intercept	9.098	0.283	32.202	0.000	275.310	0.000	0.725
	SFF	−0.024	0.045	−0.532	0.596			
	FBM	0.000	0.003	−0.135	0.893			
	FAW	−0.009	0.067	−0.141	0.889			
	RW	0.000	0.001	0.360	0.720			
	Resting	−0.051	0.010	−5.077	0.000			
	Eye‐submerging	−0.015	0.064	−0.241	0.810			
	Vigilance	−0.014	0.004	−3.081	0.003			
	RS	0.197	0.015	12.966	0.000			
	Flying	0.451	0.015	30.070	0.000			
	Diving	0.030	0.003	11.028	0.000			
Log (EI + 1)	SFF	1.597	0.178	8.999	0.000	316.180	0.000	0.278
	Diving	0.010	0.006	1.726	0.088			
	Feeding	−0.061	0.062	−0.982	0.329			
	Swimming	−0.005	0.007	−0.613	0.542			
Cube_root (NET)	Intercept	−1.301	0.509	−2.555	0.013	691.120	0.000	0.236
	SFF	2.449	0.263	9.318	0.000			
	Diving	0.014	0.009	1.636	0.106			
	Feeding	−0.130	0.092	−1.424	0.158			
	Swimming	−0.007	0.011	−0.633	0.528			

Abbreviations: DF, disturbance number; EE, energy expenditure rates; EI, energy intake rates; FAW, average fish weight; FBM, fish biomass; NET, net energy intake rates; RS, running on water; RW, river width; SFF, successful foraging frequency; TP, temperature; WD, water depth; WV, water velocity.

**FIGURE 3:**
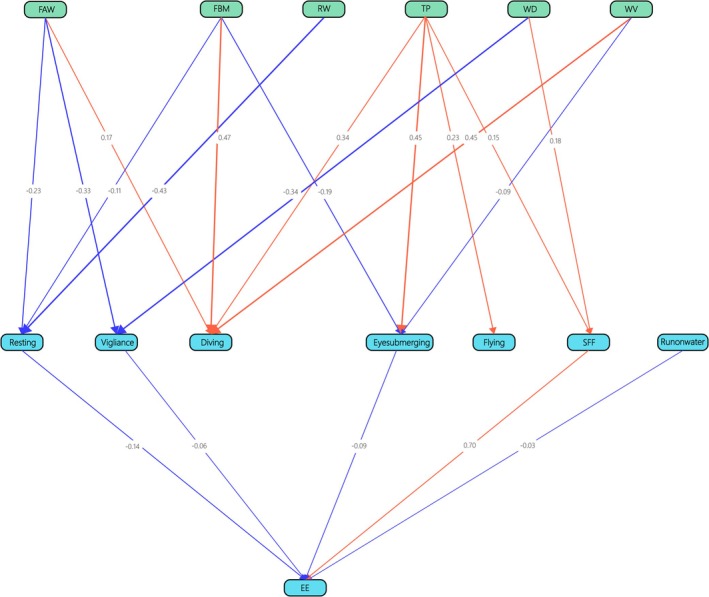
The direct and indirect influences of environmental factors and behavior changes on energy expenditure rates of Chinese mergansers. The blue and red lines denote negative and positive relationships, respectively, while the numerical values indicate the weight value of the factors. Resting, vigilance, eye‐submerging, running on water and SFF had relationships with EE; however, environmental factors did not influenced EE via behaviors in SEM. EE, energy expenditure rates; FAW, average fish weight; FBM, fish biomass; RW, river width; TP, temperature; WD, water depth; WV, water velocity. *χ* ^2^ = 112.046, df = 45, *p* = 0.000, RMSEA = 0.131, SRMR = 0.088, and CFI = 0.915.

We obtained a model *χ* ^2^ of 112.046 with 45 degrees of freedom and a *p*‐value of 0.000. The RMSEA, SRMR, and CFI were 0.131, 0.088, and 0.915, respectively. This indicated that average fish weight, fish biomass, temperature, river width, water depth, and water velocity had no impact on energy expenditure rates through resting, vigilance, diving, flying, running on water, eye‐submerging duration, and successful foraging frequency (Figure 3; Additional file 3).

Finally, running on water, flying, and diving increased energy expenditure rates, whereas vigilance and resting decreased energy expenditure rates. The model was significantly better than the null model (*χ* ^2^ = 275.310, *p* = 0.000), and the Pseudo *R* ^2^ was 0.725, indicating that the model explained approximately 72.5% of the variation (pseudo *R* ^2^ = 0.725; Table 5).

### 3.3 Relationship Between Energy Intake Rates and Environmental Factors

The energy intake rates of Chinese mergansers were 55.240 ± 112.056 J/s. Energy intake rates associated with successful foraging frequency (*r* = 0.606, *p* = 0.000), foraging (*r* = 0.347, *p* = 0.001), diving (*r* = 0.274, *p* = 0.001), and feeding time (*r* = 0.258, *p* = 0.016) showed a positive correlation but were negatively correlated with swimming (*r* = −0.267, *p* = 0.013). Feeding had a positive relationship with river width (*r* = 0.214, *p* = 0.046) and fish biomass (*r* = 0.436, *p* = 0.000) but had a negative relationship with water velocity (*r* = −0.217, *p* = 0.043), temperature (*r* = −0.273, *p* = 0.011), and disturbance number (*r* = −0.214, *p* = 0.047); however, only river width and temperature, and disturbance number significantly led to changes in feeding time. The model was significantly better than the null model (*χ* ^2^ = 23.614, *p* = 0.000), and the Pseudo *R* ^2^ was 0.228 (Table 5). Swimming negatively correlated with fish biomass (*r* = −0.238, *p* = 0.026) and successful foraging frequency (*r* = −0.391, *p* = 0.000), and fish biomass did not lead to changes in swimming time. The model was similar to the null model (*χ* ^2^ = 4.381, *p* = 0.065) (Table 5). Energy intake rates showed no relationship with fish biomass, temperature, water velocity, river width, water depth, disturbance time, disturbance distance, and disturbance number (Additional file 2).

**FIGURE 4:**
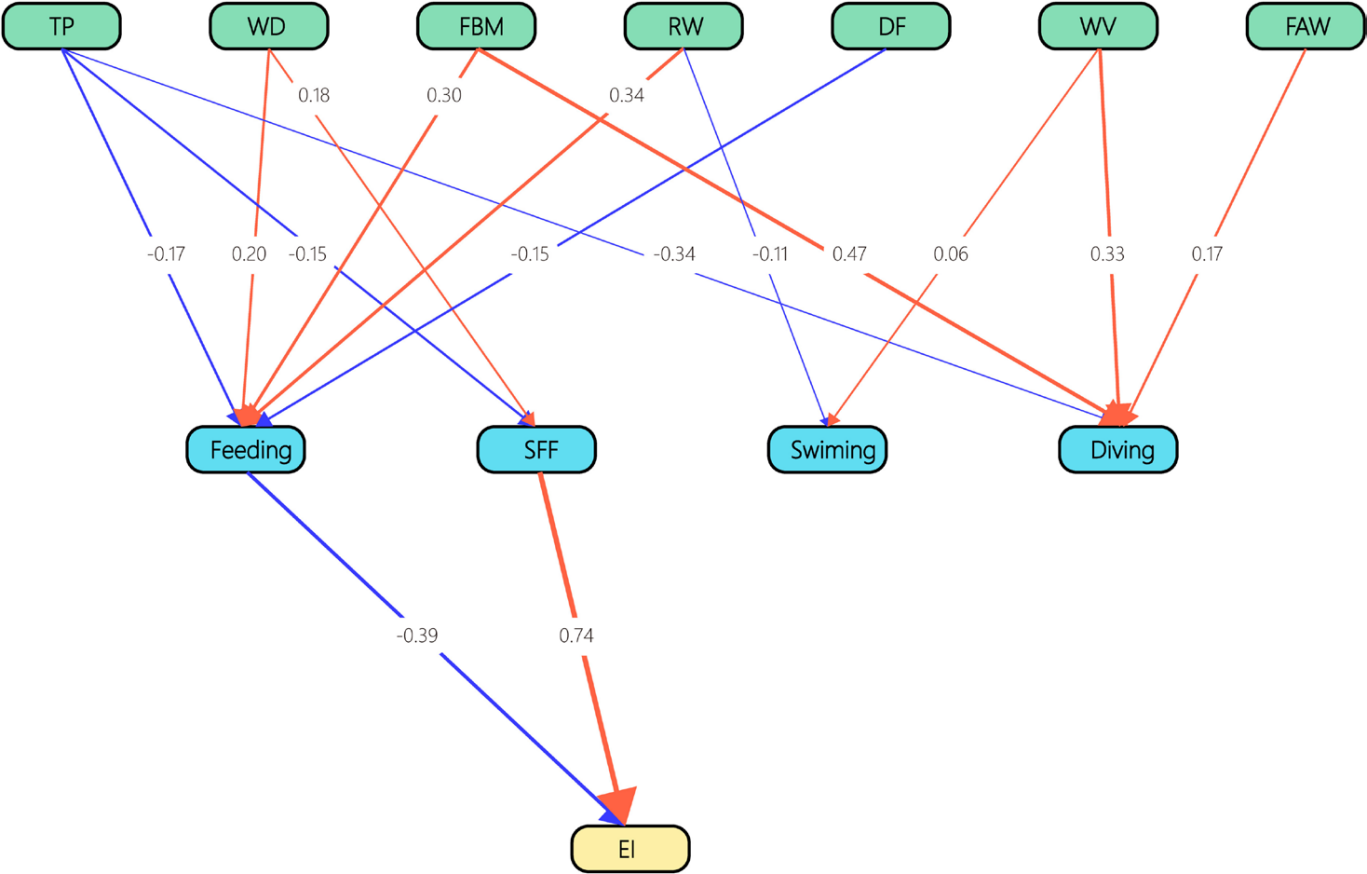
The direct and indirect influences of environmental factors and behavior changes on energy intake rates of Chinese mergansers. The blue and red lines denote negative and positive relationships, respectively, while the numerical values indicate the weight value of the factors. Feeding and SFF had relationships with EI; however, environmental factors did not influenced EI via behaviors in SEM. DF, disturbance number; EI, energy intake rates; FAW, average fish weight; FBM, fish biomass; RW, river width; SFF, successful foraging frequency; TP, temperature; WD, water depth; WV, water velocity. *χ* ^2^ = 165.888, df = 29, *p* = 0.000, RMSEA = 0.233, SRMR = 0.158, and CFI = 0.477.

We obtained a model *χ* ^2^ of 165.888 with 29 degrees of freedom and a *p*‐value of 0.000. The RMSEA, SRMR, and CFI were 0.233, 0.158, and 0.477, respectively. This indicated that fish biomass, river width, water velocity, average fish weight, water depth, temperature, and disturbance number had no impact on energy intake rates through successful foraging frequency, diving, swimming, and feeding time percentage (Figure 4; Additional file 3).

Successful foraging frequency increased energy intake rates. The model was significantly better than the null model (*χ* ^2^ = 316.180, *p* = 0.000), and the Pseudo *R* ^2^ was 0.278, indicating that the model explained approximately 27.8% of the variation (pseudo *R* ^2^ = 0.278; Table 5).

### 3.4 Relationship Between Net Energy Intake Rate and Environmental Factors

The net energy intake rate of Chinese mergansers was 45.327 ± 111.613 J/s. The increase in net energy intake rate was positively correlated with successful foraging frequency (*r* = 0.604, *p* = 0.000), foraging (*r* = 0.344, *p* = 0.001), diving (*r* = 0.267, *p* = 0.012), and feeding time (*r* = 0.256, *p* = 0.017) but negatively correlated with swimming time (*r* = −0.265, *p* = 0.013). Average fish weight, fish biomass, temperature, water velocity, river width, water depth, disturbance distance, frequency, and duration showed no correlation (Additional file 2).

**FIGURE 5:**
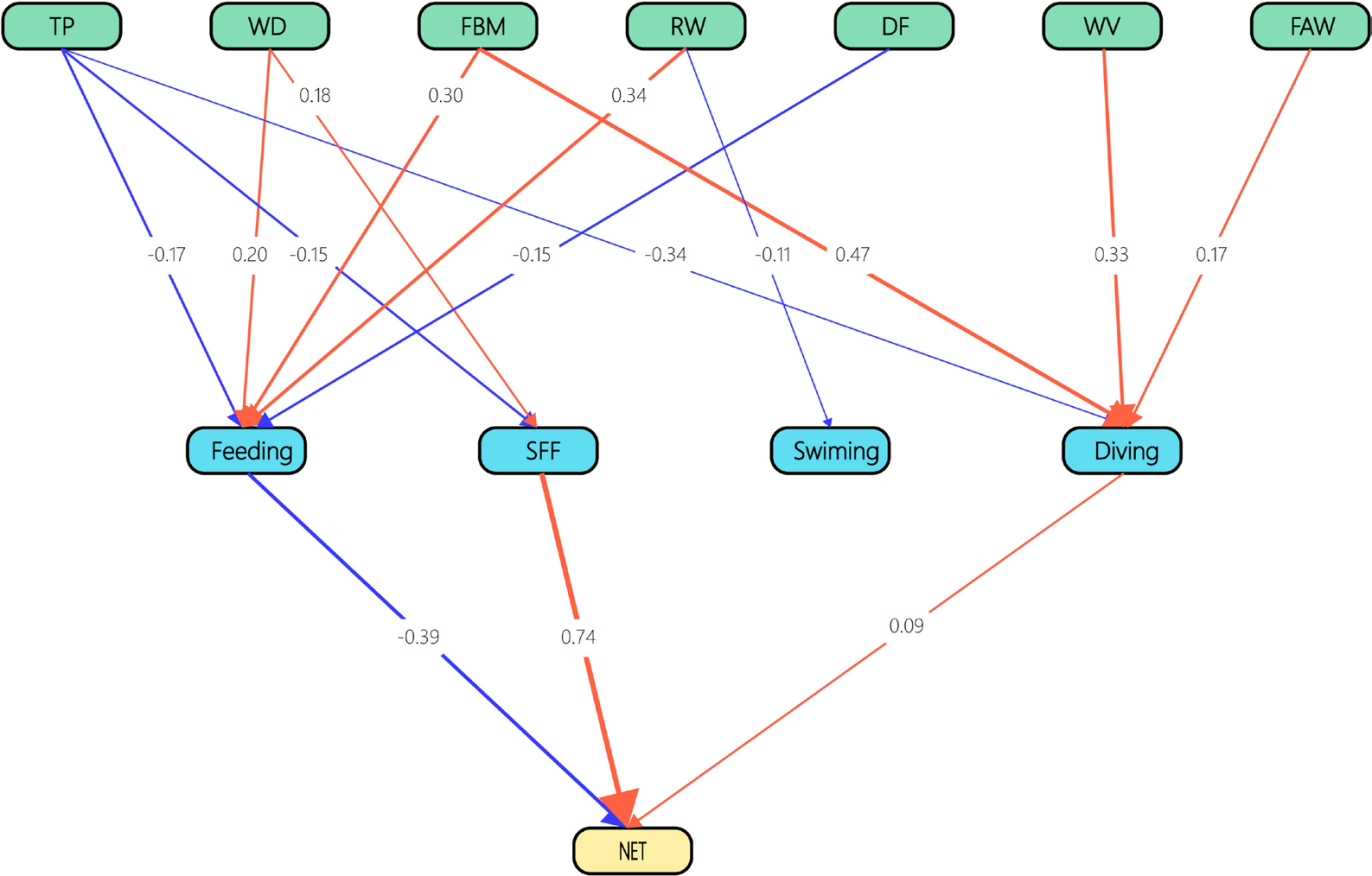
The direct and indirect influences of environmental factors and behavior changes on net energy intake rates of Chinese mergansers. The blue and red lines denote negative and positive relationships, respectively, while the numerical values indicate the weight value of the factors. Feeding and SFF had a relationship with NET, however, environmental factors did not influenced NET via behaviors in SEM. DF, disturbance number; FAW, average fish weight; FBM, fish biomass; NET, net energy intake rates; RW, river width; SFF, successful foraging frequency; TP, temperature; WD, water depth; WV, water velocity. *χ* ^2^ = 165.895, df = 29, *p* = 0.000, RMSEA = 0.233, SRMR = 0.158, and CFI = 0.476.

We obtained a model *χ* ^2^ of 165.895 with 29 degrees of freedom and a *p*‐value of 0.000. The RMSEA, SRMR, and CFI were 0.233, 0.158, and 0.476, respectively. These results indicate that fish biomass, river width, water velocity, average fish weight, water depth, temperature, and disturbance number had no impact on energy intake rates via successful foraging frequency, diving, swimming, and feeding time percentage (Figure 5; Additional file 3).

We analyzed the relationship between the net energy intake rate and influencing factors. The results showed that successful foraging frequency increased net energy intake rates. The model was significantly better than the null model (*χ* ^2^ = 391.120, *p* = 0.000), and the Pseudo *R* ^2^ was 0.236, indicating that the model explained approximately 23.6% of the variation (pseudo *R* ^2^ = 0.236; Table 5).

## 4 Discussion

This study shows that for Chinese mergansers to achieve higher energy intake, their energy costs will increase. Mergansers adjusted their behavioral time allocations to regulate energy intake, expenditure, and net intake rate, largely independent of environmental factors, except for energy expenditure rate and average fish weight, fish biomass, river width, and successful foraging frequency. Nevertheless, these environmental factors did not affect energy levels via behavioral changes.

### 4.1 Energy Expenditure Rates

The energy expenditure rate of the mergansers was determined based on the duration of time spent running on water, flying, diving, and eye‐submerging. The disturbance distance, frequency, and time were not correlated with the energy expenditure rate and behaviors, excluding feeding and resting time, which is consistent with the results of a previous study (Yu et al. 2024). This may be due to the number of launderers and fishermen in the Huangshan rivers, which are non‐lethal human disturbances that waterbirds adapt to (Béchet et al. 2004; Yu et al. 2019a).

Chinese mergansers prefer wide rivers with high water quality and low‐flowing water (Mei et al. 2018; Shao et al. 2015). This is because waterbirds invest more time foraging and less time resting and vigilant in wide rivers and low‐flowing water. These results aligned with previous studies (Zeng et al. 2015a; Yi et al. 2019). However, energy expenditure decreases diving (3.2) and increases head‐dipping (2.5) in fast‐flowing water, and thus, the energy expenditure rate was unchanged.

Chinese mergansers must consume more food to supplement energy expenditure in cold weather (Zeng et al. 2013). When the temperature decreases, mergansers increase their successful foraging frequency with full‐water diving and less eye‐emerging foraging. Consequently, the mergansers feed more when catching larger fish with full‐water diving and focus less on eye‐emerging foraging for lower fish biomass.

### 4.2 Energy Intake Rates

Energy intake rates were positively correlated with foraging time, successful foraging frequency, and feeding time. This indicates that greater foraging and feeding time and successful foraging frequencies will increase the energy intake of Chinese mergansers. The primary food source for Chinese mergansers during the winter season is fish (Zeng et al. 2015a, b). The foraging composition, that is, diving, successful foraging frequency, and feeding, which was found foraging time allocation in waterbirds during the non‐breeding period, is closely related to the quality and availability of food and individual energy requirements (Nasser and Boudjéma 2008). The more time waterbirds devote to foraging, the more food they obtain (Yu et al. 2019a, b), and the frequency of successful foraging events directly affects their food and energy intake rates.

Foraging for Chinese mergansers is influenced by habitat conditions, as lower temperatures reduce foraging efficiency (Zeng et al. 2015b). This study revealed an increase in successful foraging frequency or diving behavior with decreasing temperature. This correlation was due to the fish concentration and increased biomass at lower temperatures, which could further influence foraging behavior. Foraging behaviors that positively affect energy intake increased at lower temperatures; however, diving decreased and eye‐emerging increased. Energy intake rates did not change with temperature.

Increased flow rates have previously been found to reduce the foraging efficiency of Chinese mergansers (Zeng et al. 2015b). Our study results did not agree with this, and this may be because the Chinese mergansers found suitable feeding areas in the rivers; however, the energy intake rate did not change due to reduced fish biomass. The Chinese mergansers devoted more time to eye‐emerging when flow rates were increased.

The energy intake rate was unrelated to the water depth because the mergansers increased resting and successful foraging frequency rather than foraging time. Zeng et al. also reported this phenomenon (Zeng et al. 2015b), in which water depth significantly negatively affected the appearance of Chinese mergansers because it is not conducive to diving.

### 4.3 Net Energy Intake Rates

The net energy intake rate of Chinese mergansers is significantly correlated with their energy intake rate, as confirmed in previous studies (Wu et al. 2014). The net energy intake rate was also positively correlated with successful foraging frequency and negatively correlated with feeding time, indicating that more successful foraging increases the energy balance in Chinese mergansers but not feeding time. The net energy intake rate was not related to water depth, water velocity, or river width, and the same effects for behaviors and fish. As water depth increases, mergansers are observed to have more foraging success and rest; thus, the effects of energy intake and resting expenditure are counterbalanced. When the water velocity increased, eye‐submerging and head‐dipping foraging increased, whereas diving decreased. These changes may compensate for the resulting lower food intake, as previously reported for the Greater White‐fronted Goose (Fan et al. 2020). When the river width and overall foraging and fish biomass increased, the rest and vigilance times were reduced; however, the successful foraging frequency remained unchanged, indicating that energy intake effectively compensated for the energy expended during foraging.

A positive relationship was observed between the net energy intake rate and the rate of energy expenditure. This finding is inconsistent with the OFT and previous studies (Mizrahy‐Rewald et al. 2022), which indicated that wintering Chinese mergansers forage and rest in rivers at most times of the day, and the resulting energy expenditures are less than those of flying. Birds employ a variety of energy‐balancing strategies; the more they invest, the greater the energy balance (Wu et al. 2014). The net energy intake rate was primarily affected by successful foraging frequency and feeding time. This occurs because the greater the successful foraging frequency, the more energy they can provide. When diving for fish increases the successful foraging frequency, all behaviors not related to foraging decrease, which means that mergansers put all their energy into foraging, increasing their energy intake and reducing unnecessary energy expenditure except for foraging. This resulted in a significant increase in energy intake. When the temperature and water depth decreased, the mergansers used this foraging mode more frequently.

### 4.4 Limitations and Future Studies

This study has several limitations. First, Chinese mergansers are timid and easily frustrated, making it difficult to locate and record them. In future investigations, the number of sampling sites and collected samples should be increased. Second, this study was limited to winter. In future studies, data should be obtained during the summer to achieve the annual cycle and help elucidate the energy balance strategy of Chinese mergansers throughout their life cycle.

## 5 Conclusions

This study investigated the influence of behavioral and microhabitat variables on the energy balance patterns of Chinese mergansers. The results showed varying energy intake, expenditure, and net energy intake rates in response to changes in heterogeneous rivers. Chinese mergansers increased energy intake or net intake with increased energy expenditure rates; alternatively, they adjusted their behavioral time allocations to regulate these rates, largely independent of environmental factors, except for energy expenditure rates and average fish weight, fish biomass, river width, and successful foraging frequency. The behaviors influencing the energy balance were modified in response to environmental factors. However, environmental factors did not affect the energy levels through behaviors. The key factors influencing net energy intake were successful foraging frequency and feeding time percentage. This study enhances our understanding of energy balance patterns in endangered wintering waterbirds and the mechanisms of energy balance in Chinese mergansers. Moreover, the results provide information that can be used to guide habitat conservation and management and thus support the stability of wintering populations.

## Author Contributions

**Chao Yu:** conceptualization (equal), formal analysis (equal), funding acquisition (equal), investigation (equal), writing – original draft (equal). **Hao Zheng:** data curation (equal), formal analysis (equal), writing – original draft (equal). **Yu Luo:** investigation (equal), software (equal). **Mengxue Guo:** investigation (equal), visualization (equal). **Shiqi Wang:** investigation (equal), methodology (equal). **Xuanshuo Qi:** investigation (equal). **Qun Li:** investigation (equal). **Zhonghai Lv:** conceptualization (equal), investigation (equal), software (equal), visualization (equal), writing – review and editing (equal).

## Conflicts of Interest

The authors declare no conflicts of interest.

## Data Availability

The data that support the findings of this study are openly available at https://github.com/xiaoyuhuoying/Chinese‐mergansers/issues.
